# Oxidative stress and COVID-19-associated neuronal dysfunction: mechanisms and therapeutic implications: SARS-CoV-2 (COVID-19)-mediated oxidative stress in neurons

**DOI:** 10.3724/abbs.2023085

**Published:** 2023-06-25

**Authors:** Dylan R. Bowen, Suhrud Pathak, Rishi M. Nadar, Rachel D. Parise, Sindhu Ramesh, Manoj Govindarajulu, Austin Moore, Jun Ren, Timothy Moore, Muralikrishnan Dhanasekaran

**Affiliations:** 1 Department of Drug Discovery and Development Harrison College of Pharmacy Auburn University Auburn-AL 36849 USA; 2 Department of Cardiology Zhongshan Hospital Fudan University Shanghai 200032 China; 3 Department of Laboratory Medicine and Pathology University of Washington Seattle WA 98195 USA

**Keywords:** COVID-19, oxidative stress, antioxidants, cytokine storm, neuronal dysfunction

## Abstract

Severe acute respiratory syndrome (SARS)-CoV-2 virus causes novel coronavirus disease 2019 (COVID-19), and there is a possible role for oxidative stress in the pathophysiology of neurological diseases associated with COVID-19. Excessive oxidative stress could be responsible for the thrombosis and other neuronal dysfunctions observed in COVID-19. This review discusses the role of oxidative stress associated with SARS-CoV-2 and the mechanisms involved. Furthermore, the various therapeutics implicated in treating COVID-19 and the oxidative stress that contributes to the etiology and pathogenesis of COVID-19-induced neuronal dysfunction are discussed. Further mechanistic and clinical research to combat COVID-19 is warranted to understand the exact mechanisms, and its true clinical effects need to be investigated to minimize neurological complications from COVID-19.

## Introduction

In a general sense, oxidative stress is induced by an imbalance in antioxidants and reactive oxygen and nitrogen species (RONS). RONS serve some purposes in the body, such as cell signaling, but when the body loses the ability to detoxify them at a sufficient rate, these free radical species start to cause cell and tissue damage [1]. Common RONS that are produced in the body as a result of normal metabolic processes are the superoxide anion (O _2_^–^), hydrogen peroxide (H _2_O _2_), hydroxyl radicals (HO **^.^**), nitric oxide (NO **^.^**), peroxynitrite (ONOO ^–^), hypothiocyanite (OSCN ^–^), and hypochlorous acid (HOCl) [2]. Exogenous sources of RONS include inhaling polluted air, drinking polluted water, drugs, alcohol, certain types of foods (especially fatty foods and smoked meats), radiation, and excessive amounts of certain heavy and transition metals [3]. The main producers of RONS in the body are mitochondria, xanthine oxidoreductase (XOR), reduced nicotinamide adenine dinucleotide phosphate (NADPH) oxidases, peroxidases, and nitric oxide synthases [2].

Reactive oxygen species (ROS) are generated in the electron transport chain, mostly by Complex I but also by Complexes II and III, in mitochondria because of acute hyperoxia or hypoxia, which cause the production of the superoxide anion and hydrogen peroxide [4]. Although XOR is involved in multiple essential pathways, such as inflammation, repair, aging, cell growth, cell differentiation, cell mobility, and endothelial and vascular regulation, it is also involved in pathological conditions such as gout, renal diseases, and cardiovascular diseases. This involvement is because it is a major producer of superoxide ions, hydrogen peroxide, and nitric oxide [5]. NADPH oxidases (NOX) convert diatomic oxygen and NADPH to the superoxide ion and NADP ^+^. It is present in large amounts in phagocytes [6]. This presence in phagocytes connects this enzyme to its role in initial inflammatory responses, such as in the insulin response, and then, with excess activity, the pathogenesis of inflammatory diseases, such as obesity, preeclampsia, hypertension, aortic media hypertrophy, atherosclerosis, diabetic complications, atrial fibrillation, cardiac hypertrophy, ischemic stroke, Alzheimer’s Parkinson’s, HIV dementia, many types of cancer, liver ischemia, diabetic nephropathy, pulmonary fibrosis, chronic obstructive pulmonary disease, asthma, and possibly other diseases characterized by RONS generation [7]. Peroxidases are in multiple locations throughout the body. Their general function is to oxidize substrates in the presence of hydrogen peroxide. They can create oxidants such as nitric dioxide (NO _2_), hypothiocyanite (OSCN ^–^), superoxide ions, and hypochlorous acid (HOCl), which are all powerful oxidants [2]. Peroxisomes create these strong oxidants as antimicrobial agents used to fight infections via bacteria, parasites, and viruses. Eosinophil peroxidase is specifically linked to the hypersensitive response by the immune system in allergic reactions, thus contributing to peroxidase’s role in pathology in the body [8].

Inducible nitric oxide synthase [9] is linked to cerebral ischemia and hypoxia, and the nitric oxide synthase/nitric oxide pathway is connected to all forms of vascular dementia. This is caused by pathological conditions, aging, and vascular risk factors, which inactivate endothelial NOS (eNOS). This inactivation prevents normal NO production, reduces NO bioavailability, triggers cerebrovascular endothelial cell (CEC) dysfunction, and overactivates neuronal NOS (nNOS) and iNOS, creating an excessive amount of NO that leads to neurotoxicity and neuroinflammation [8]. Traditionally, four main biological changes have been used to define and encapsulate what oxidative stress means in the body. These are, namely, the detection of RONS generation, a decrease in antioxidant activity, biomarkers of oxidative stress such as lipid peroxidation and DNA oxidation, and a disturbance of cellular redox status [10]. Inflammation is a major marker of oxidative stress. Leukocytes and mast cells undergo an increase in respiration rates in damaged regions and therefore produce and release more RONS. These inflammatory cells produce mediators such as cytokines that can initiate a cascade of events that reduce the body’s ability to reduce RONS and create a negative feedback loop in which inflammation is induced by oxidative stress [11]. Inflammation with oxidative stress also reflects the body’s response to an infection. The body generates superoxide, hydrogen peroxide, and hydrochloric acid at infection sites to try and kill invading pathogens. Escalation and excessive production of inflammatory mediators such as tumor necrosis factor-α (TNF-α), interleukins 1 and 6 (IL-1, IL-6), and the activation of the nuclear factor kappa B (NF-κB) pathway can, unfortunately, cause a vicious cycle of RONS overproduction and inflammation that drive each other [10]. It is important to note that even though these things are generally markers for and measurements of oxidative stress, the body has a continuously changing balance between RONS production and antioxidant activity, so multiple factors should be analysed to determine oxidative stress to not confuse changing levels with the body’s changing homeostasis [12]. Oxidative stress is thought to be involved in the development and progression of many age-related diseases, such as cardiovascular disease, chronic kidney disease, acute kidney disease, neurodegenerative diseases including Alzheimer’s and Parkinson’s disease, macular degeneration, biliary diseases, diabetes mellitus, chronic obstructive pulmonary disease (COPD), and cancer [3]. The function of oxidative stress in relation to SARS-CoV-2 and the underlying processes associated with neuronal dysfunction are discussed in this study.

## COVID-19 and Cytokine Storm

According to the World Health Organization (WHO), there have been over 430 million cases of novel coronavirus disease-19 (COVID-19) and almost 6 million cumulative deaths caused by COVID-19. According to the WHO, in the last 24 hours alone, there have been over 1.6 million new cases of COVID-19 reported. Thankfully, over 10 billion vaccine doses have been administered globally, according to the WHO. The COVID-19 pandemic caused by severe acute respiratory syndrome coronavirus-2 (SARS-CoV-2) is still raging years after it started, even with the release of vaccines and the employment of social distancing. This pandemic, which began in Wuhan, China, and originated in bats, is the largest and longest pandemic for the lives of the majority of the globe [13]. The continuation of the pattern and the surges of infections from SARS-CoV-2 come from both its ability to infect many individuals quickly and its high rate of mutation. Many variants of this novel coronavirus include Alpha, Beta, Delta, Gamma, Epsilon, Eta, Iota, Kappa, Mu, Zeta, and Lambda. Of major concern recently was the deadlier Delta variant [14]. The Omicron variant is currently the most concerning variant because of its higher infection rates when compared to previous variants. There is also concern that the Omicron variant will still cause disease in people who have been vaccinated against the SARS-CoV-2 virus due to its mutations away from the original disease [15].

COVID-19 induces a cytokine storm in the body that is related to the severity of the disease. These cytokine storms are the manifestations within the body that cause severe effects that those infected by COVID-19 feel. In excess of the production of these cytokines, some of those infected can experience widespread tissue damage, multiorgan failure, and even death [16]. SARS-CoV-2 enters the cell through the binding of its surface S proteins, which give the virus a crown-like appearance, to the angiotensin-converting enzyme 2 (ACE2) receptor, and the S protein anchors the virus to the surface of cell membranes [ 17– 19] . Moreover, neuropilin (NRP-1), which is a coreceptor for vascular endothelial growth factor (VEGF), integrins, and plexins, is also considered to participate as a coreceptor for SARS-CoV-2, facilitating virus entry via the olfactory epithelium. For SARS-CoV-2 to bind to angiotensin-converting enzyme 2, NRP-1 is considered to be a cofactor (ACE2) [ 20– 22] . The virus then enters the cell and uncoats its viral ribonucleic acid (RNA) into the cytoplasm, where it is processed by the endoplasmic reticulum. Then, a new virus is made and sent out of the cell by the Golgi apparatus [23]. Cytokine storm is induced by toll-like receptors (TLRs), which sense invading pathogens based on pathogen-associated molecular pattern (PAMP). Recognition of the SARS-CoV-Tho2 virus is performed by TLR4 (on the cellular membrane), TLR7, and TLR8 (both of which are endosomal). SARS-CoV-2 binds TLR4, which activates activator protein 1 (AP1), NF-κB, and IRF and regulates IL-6 via NF-κB [24]. The SARS-CoV-2 virus is recognized by TLR7 and TLR8 because its viral load is single-stranded RNA (ssRNA) [25]. TLR4 signals through both the MyD88 and Toll-IL-1 receptor-domain-inducing IFN-β (TRIF) pathways; therefore, TLR4 activates both the NF-κB and IRF signaling pathways. This activation of the two pathways contributes to the dysregulated cytokine production seen in a cytokine storm [26]. TLR7 and TLR8 activate the MyD88-dependent NF-κB pathway. This produces TNF-α and ILs. IL-6 is specifically elevated in SARS-CoV-2 patients, and IL-6 inhibits the expression of cytotoxic T cells as well as suppressor of cytokine signaling-3 (SOCS3). IL-6 also increases the expression of PD1 and therefore reduces T-cell generation and expansion [27]. When TLRs sense PAMPs, NF-κB and interferon regulatory factor (IRF) are stimulated to produce type I interferons (IFNs) [28]. Type I IFNs induce an antiviral response in the body by inhibiting viral replication in infected cells, inducing the creation and presentation of antigens, and enhancing the responses of B cells, T cells, natural killer (NK) cells, dendritic cells, and monocytes. The problems from Type I IFNs come from severe acute infections such as those seen in COVID-19. TNF-α related apoptosis-inducing ligand (TRAIL) and the CD95 ligand (CD95L) induce apoptosis of endothelial cells and lymphocytes through their own cell death receptors. TRAIL and CD95L are overexpressed by inflammatory monocytes in acute infections, thus causing more cell death and adding to the immunopathology of the SARS-CoV-2 virus. Programmed cell death ligand 1 (PDL1), when expressed due to type I IFNs, suppresses the function of T cells with programmed cell death protein 1 (PD1) [29]. Thus, type I interferons contribute to both the immunosuppression and immunopathology of acute infections as part of the cytokine storm caused by COVID-19. More research needs to be done to connect the activation of TLRs to COVID-19-induced cytokine storms as an exact mechanism, and causality has not been established to date. Protein ISGylation is a conjugated protein form of Type I IFN-inducible ubiquitin-like protein IFN-stimulated gene 15 (ISG15). ISGylation induces cytokine storms and causes colon inflammation, and patients with severe COVID-19 have a higher rate of ISGylation, which may indicate that it plays a role in the induction of cytokine storms and contributes to COVID-19 severity [30].

## Oxidative Stress-based Signal Transduction Pathway in COVID-19

**Figure 1:**
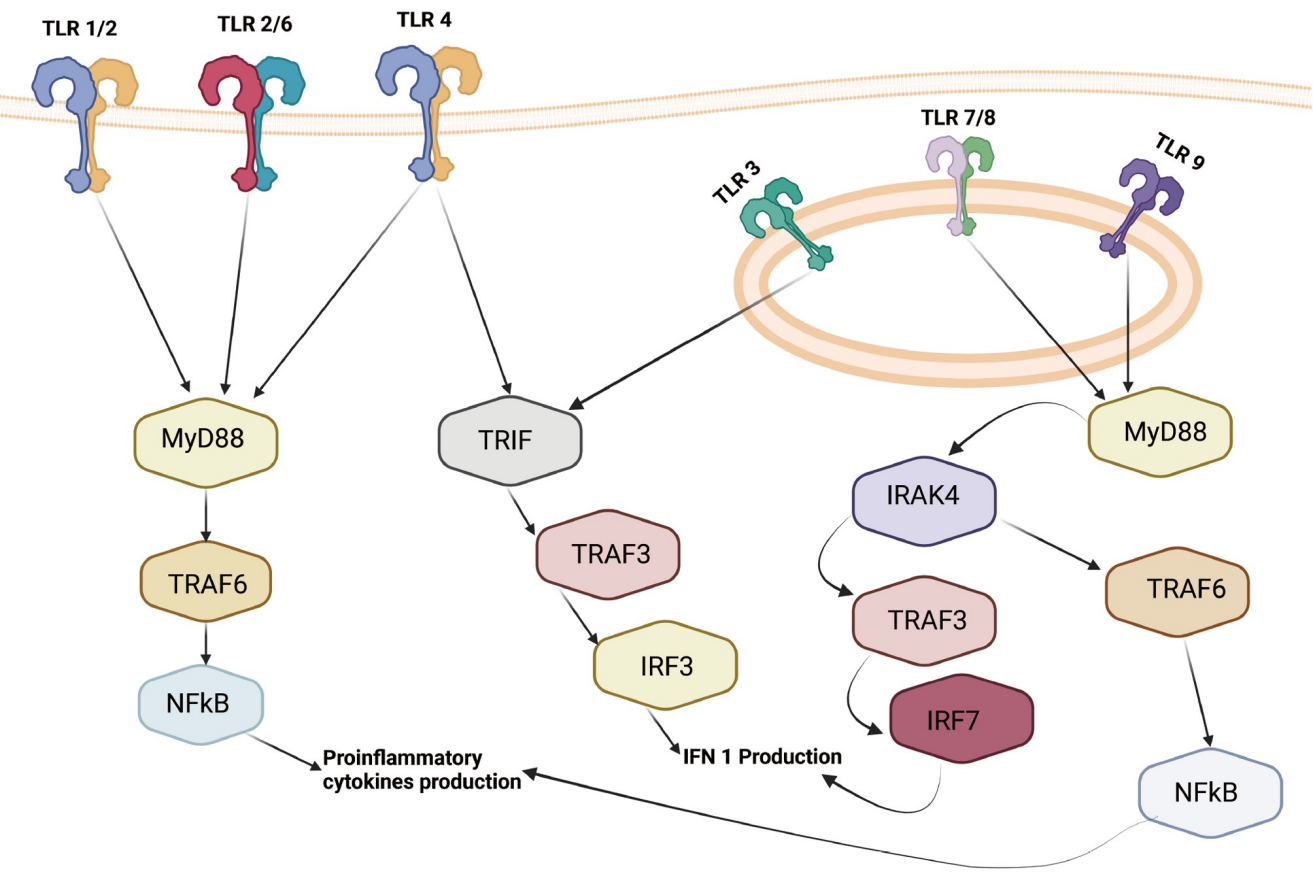
Figure 1 Toll-like receptor signaling pathways TLR1/2, TLR2/6 and TLR4 are in the cell membrane. TLR1/2 and TLR2/6 activate the MyD88 pathway, while TLR4 activates both the TRIF pathway and the MyD88 pathway. TLR3, TLR7, TLR8, and TLR9 are located inside endosomes. TLR3 activates the TRIF pathway, and TLR7, TLR8, and TLR9 activate the MyD88 pathway. The SARS-CoV-2 virus activates TLR4 prior to entering the cell, activating both the MyD88 and TRIF pathways. SARS-CoV-2 virus does this via its spike protein.

The SARS-CoV-2 virus has a receptor-binding domain (RBD), which uses the ACE2 protein as an entry receptor with the aid of its S protein, which can bind to transmembrane serine protease 2 (TMPRSS2) as well [ 31, 32] . When both ACE2 and TMPRSS2 bind to S proteins in SARS-CoV-2, the S protein is cleaved from the envelope, and this cleavage exposes the fusion peptide (FP), which is thrust into the cell membrane, initiating membrane fusion [23]. Toll-like receptors (TLRs) are type I transmembrane glycoproteins that are considered pattern recognition receptors used in the innate immune system to detect pathogen-associated molecular patterns (PAMPs) [33]. TLRs are critical to the innate immune response and play a major role in cell signaling associated with the immune response. They are located on the cell surface, intracellular compartments, and endosomes [34]. There are ten TLRs in humans that are classified into different families based on different characteristics, such as location and recognition material [35]. These TLRs are one of the first stages of a cascade of immune response events in the body [36], as shown in Figure 1 [37].

**Figure 2:**
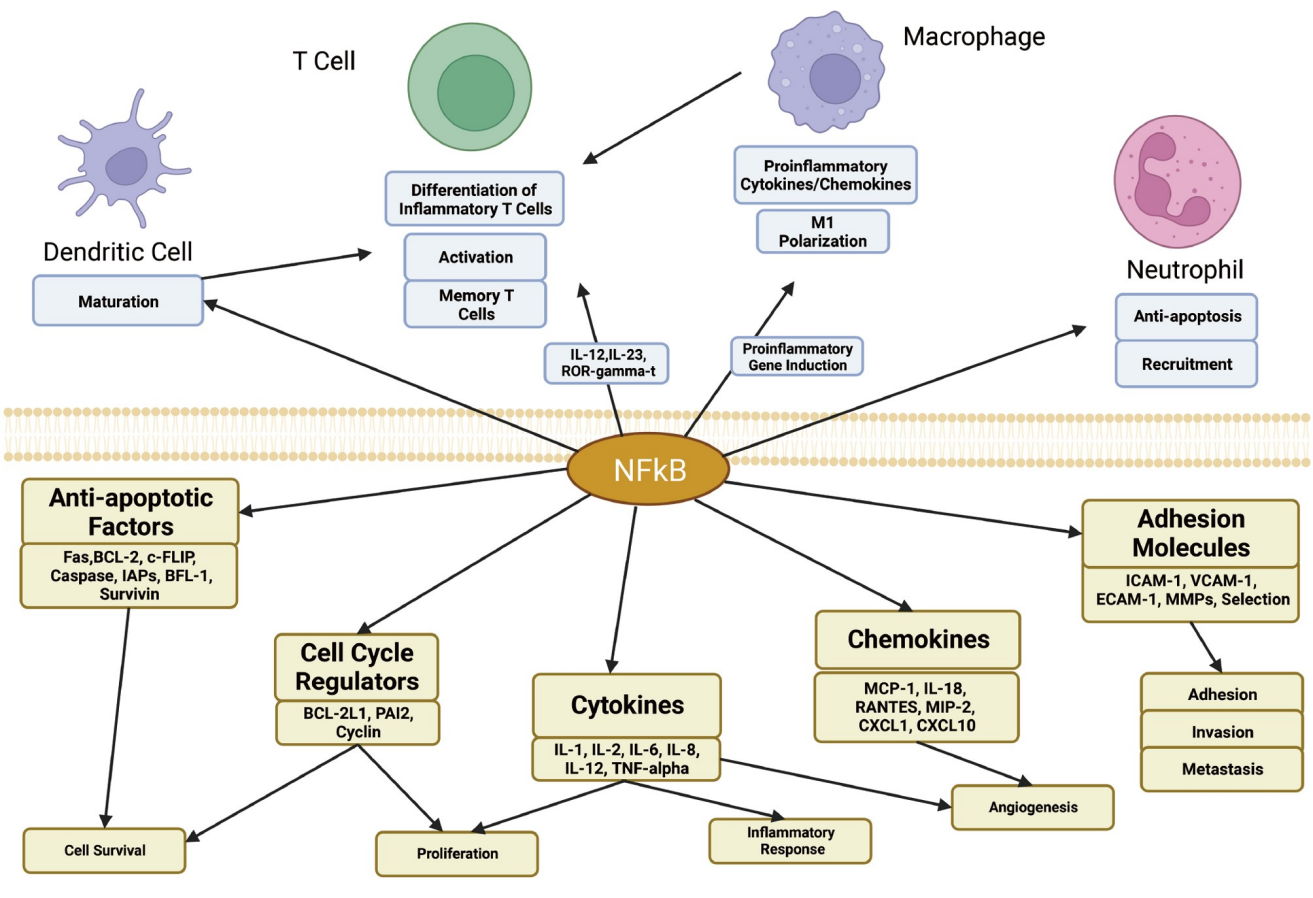
Figure 2 In response to the activation of PAMPs signaling, NF-κB triggers the transcription of a group of inducible proinflammatory target genes involved in various cellular processes

The SARS-CoV-2 virus activates TLR4 prior to entering the cell, activating both the MyD88 and TRIF pathways. SARS-CoV-2 does this via its spike protein [37]. Not pictured in Figure 1 is TLR1 (or TLR1/2), which the spike protein of SARS-CoV-2 also interacts with along with TLR6. TLR1/2 and TLR2/6 activate the MyD88 pathway [38]. As shown in Figure 1, the activation of these TLRs activates the NF-κB pathway via MyD88 and TRAF6. After SARS-CoV-2 enters the cell, its ssRNA is recognized by TLR7/8, which are in the membranes of endosomes. When viral ssRNA replicates and forms dsRNA, TLR3 is activated as well. These TLRs activate the MyD88 and TRIF pathways, which then induce the NF-κB inflammatory response as well as interferon (IFN) type I/III production [38]. The NF-κB pathway regulates inflammatory responses by inducing proinflammatory genes in response to the activation of either its canonical or noncanonical pathways. In the case of SARS-CoV-2, the canonical NF-κB pathway is activated. This pathway also regulates T cell activation and differentiation [ 39, 40] . NF-κB performs these functions through the transcriptional induction of cytokines, chemokines, adhesion molecules, anti-apoptotic factors, cell cycle regulators, and other mediators of inflammation [41], as shown in Figure 2.

Due to the SARS-CoV-2 virus activating the NF-κB pathway in multiple ways, SARS-CoV-2 can cause the overproduction of proinflammatory cytokines such as interleukin (IL)-1, IL-2, IL-6, IL-8, IL-12, and TNF-α [42]. The overproduction of these cytokines causes cytokine storms and indicates severe COVID-19 [28]. Proinflammatory cytokines have been shown to increase ROS through mitochondrial dysfunction and NADPH oxidase [43]. These proinflammatory cytokines activate certain immune cells to create more ROS to try and clear the infection [44]. NOX2, XOR, iNOS, COX-2, and other enzymes that produce ROS are all upregulated by the activation of NF-κB. ROS can also activate or inhibit the NF-κB pathway depending on where in the pathway ROS act [45]. The excessive inflammation caused by the cytokine storm that is induced in severe SARS-CoV-2 infections causes a positive yet destructive feedback loop in the immune response. The excessive inflammatory response causes an overproduction of ROS by inflammatory immune cells. This response is generated from the overactivation of the NF-κB pathway, which releases proinflammatory cytokines. The overproduction of ROS eventually causes oxidative stress. Inflammatory cells sense oxidative stress in the environment, which causes these cells to release more inflammatory cytokines and cause more inflammation. This, in turn, causes more ROS production and oxidative stress. Overall, the inflammatory response, which causes oxidative stress, causes further inflammation and oxidative stress in a vicious cycle that spreads through the body and results in multiorgan failure and severe COVID-19 [ 28, 44– 49] . SARS-CoV-2 can enter the brain via the neural-mucosal interface and travel along the olfactory tract of the central nervous system (CNS) and into the brain [50]. It has also been shown that the virus enters the CNS through the blood-brain barrier (BBB) via endothelial cells [51]. After entry into the CNS, SARS-CoV-2 invades CNS cells via the same mechanisms as listed previously, namely, via ACE2. This invasion then coincides with the same results as mentioned previously. These results are the overactivation of inflammatory pathways that cause oxidative stress, which causes more inflammation and more oxidative stress in a vicious feedback cycle [52]. Oxidative stress and proinflammatory cytokines have previously been linked to demyelination and axonal damage in a neuroinflammatory model [53]. Iron dysregulation has been linked to hyperinflammatory states and is the cause of ROS production in these hyperinflammatory states. This same iron dysregulation has been observed in severe COVID-19 patients [ 54, 55] . Mitochondrial dysfunction as well as the iron dysregulation that is resulted from SARS-CoV-2 infection are possible oxidative stress-based mechanisms behind brain fog and the resulting neurological disorders seen in COVID-19 because mitochondrial dysfunction and iron dysregulation, which cause oxidative stress, have been directly linked to neurodegenerative disorders such as Alzheimer’s and Parkinson’s diseases [ 56– 59] . Severe COVID-19 has previously been directly linked to Alzheimer’s disease. Once it invades the cognitive centers of the brain, SARS-CoV-2 was shown to induce Alzheimer’s-like phenotypes and an exacerbation of Alzheimer’s pathology [52].

## Markers of Oxidative Stress in COVID-19

The effects of COVID-19 on various oxidative stress markers are summarized in Table 1. Glial fibrillary acidic protein (GFAp) is usually undetectable in healthy individuals. However, when it is detected, GFAp is considered a nonspecific marker for neuropathogenesis. Studies showed that patients presenting severe cases of COVID-19 have higher levels of GFAp, thus serving as a nonspecific marker of neuropathogenesis that is correlated with delirium, neural dysfunction, and an increased risk of astrocyte damage [60].

**Table:** **Table 1** Oxidative stress markers and COVID-19

Marker	Roles in COVID-19 induced pathology
3-Nitro-tyrosine	No studies to date.
4-HNE	Possible relationship associates the cytokines, oxidative stress, and vascular stress in COVID-19. 4-HNE is increased in various vital organs [61].
8-OHdG	Significantly increase in urine in patients exposed to sodium hypochlorite during the COVID-19 [62]. Significantly increase in the plasma/blood/Urine/Serum of COVID-19 patients [ 63– 65] . Significantly increase in the Placenta during gestation [66].
Advanced glycation end-products	COVID-19 affects the receptor for advanced-glycation end-products (RAGE) [ 67, 68] . Significantly increase the products in serum [ 69– 72] .
Advanced oxidation protein products	A significant drop is observed in advanced oxidation protein products content at 7 days upon admission. In contrast, 14 days upon admission, and a significant increase is examined in advanced oxidation protein products’ levels in plasma [ 64, 73] .
Arylesterase	COVID-19 PON1 (serum paraoxonase and arylesterase) has been showing an increase in severe patients [74].
Catalase	Catalase activity significantly decreases in the Placenta and the serum [ 66, 75] .
Divalent metal	Copper: serum copper level is raised in pregnant women with COVID-19, urinary concentration of copper was higher in severe patients of COVID-19 [ 76, 77] . Zinc: serum zinc level drops in COVID-19 pregnant patients [76]. Iron: the COVID virus attacks and devastates hemoglobin, consequently causing the release of iron from porphyrins and discharging it into the circulation with significant iron overload [78]. Manganese: in severe patients, the urinary concentration of manganese is noticed to be greater as compared to the non-severe cases with COVID-19 [77]. Magnesium: serum magnesium level is enhanced during pregnancy in the COVID-19 group [76]. Calcium: calcium levels in patients with COVID-19 are significantly lower than in healthy individuals [79].
Isoprostane	Isoprostane content is found to be high in the plasma [ 63, 66] .
Glutathione (Oxidized/Reduced)	Covid-19 patients with moderate and severe symptoms show lower content of glutathione reduced [80, 81].
Glutathione peroxidase	Patients of COVID-19 show the low enzymatic activity of the Glutathione Peroxidase in the blood [82].
Glutathione reductase	Positive-PCR COVID-19 patients show a reduction in the serum activity of Glutathione Reductase [83].
GST	Glutathione S-transferase polymorphisms are linked with a higher risk of oxidative stress, which possibly plays a significant role in vulnerability to infection with COVID-19 and its consequence COVID-19 patients with GSTT1 ^−/−^ genotype have a greater risk of mortality and poorer overall survival. The concept suggests that oxidative stress is more predominant in patients with low or no Glutathione S-transferase activity. Individuals with the GSTT1 ^−/−^ genotype had a higher risk of COVID-19 infection as compared to GSTM1 ^+/+^ [ 84, 85] .
Lipid peroxide	Oxidative cell damage Lipid peroxidation [86] level was significantly higher in COVID-19 patients [87].
Lipoxygenase	Lipoxygenase enzymes are implicated in numerous processes that connect to aggravate the hyper-inflammatory conditions in SARS-CoV-2 infection [88].
Malondialdehyde	Significantly increased in urine in patients exposed to sodium hypochlorite during the COVID-19 [62, 89].
Melatonin	Melatonin, in mitochondria, possibly prevents the intracellular attack of Covid-19 by indirectly controlling the expression of angiotensin-converting enzyme 2 (ACE2), which are receptors of Covid-19 [90].
Micronutrients	Vitamin A: vitamin A deficiency can occur in SARS-CoV-2 infection [91]. Vitamin C: critical COVID-19 patients have low levels of Vitamin C [92]. Vitamin D: vitamin D deficiency occurs in patients with COVID-19 [93]. Vitamin E: in COVID-19 patients, vitamin E levels are found to be lower [94]. Carotenoids: siphonaxanthin inhibits SARS-CoV-2 entry *in vitro*. It is also a powerful anti-inflammatory and antioxidant [95]. Various classes of polyphenolic compounds: polyphenols could utilize their anti-SARS-CoV-2 potential in several ways; though, no human studies to date on the effects of polyphenolic compounds on coronavirus [96]. Coenzyme Q10: virus-induced oxidative stress results in primary CoQ10 deficiency [97].
Myeloperoxidase	Plasma myeloperoxidase-DNA complex activity is increased in COVID-19 patients [98].
NQO1	NADPH oxidase activity is increased in COVID-19 patients [99].
Nitric oxide synthase	With elevated cytokines, nitric oxide synthase activity is increased [100].
Nitrite	Lower serum nitrite content is found in the COVID-19 patients [101].
Paraoxonase	COVID-19 PON1 (serum paraoxonase 1) has been demonstrated to increase protein levels from non-severe to severe patients [74].
Peroxynitrite	The overproduction of peroxynitrite exacerbates the severity of the COVID-19 [102].
ROS	*In vitro* studies have found that Covid-19 infection increases ROS production in human promonocyte cells and various mammalian cells [103].
Superoxide anion	Severe SARS-CoV-2 patients’ levels of superoxide anion radicals were significantly higher [104].
Superoxide dismutase	Antioxidant enzymes Superoxide dismutase [105] activity was significantly higher in COVID-19 patients [ 75, 87] .
Thioredoxin reductase	Covid-19 targets TXNRD1 for a proteolytic knockdown, and in infected cells, the resulting diminishes in this vital antioxidant molecule, impacting increased oxidative stress [106].
Xanthine oxidase	Expression of Xanthine oxidase is stimulated by SARS-CoV-2 [107].

4-HNE, 4-hydroxy-2-nonenal; 8-OHdG, 8-hydroxydeoxyguanosine; GST, glutathione S-transferase; NQO1, NAD(P)H:quinone oxidoreductase 1; ROS, reactive oxygen species.

The receptor for advanced glycation end-products (RAGE) and advanced glycation end-products (AGE) together produce a signaling pathway that causes the activation of NADPH oxidase, leading to neuronal injury [108]. HMGB1 and RAGE are overexpressed in COVID-19 patients and correlated with COVID-19 severity. Together with high mobility group box protein 1 (HMGB1), RAGE signaling causes a cascade in COVID-19 pathogenesis leading to increased ROS production via NADPH oxidase activation as a cause of neuropathogenesis [109]. SARS-CoV-2 is also linked to an upregulation of cyclooxygenase-2 (COX-2), which creates metabolites that induce hyperinflammation and coagulopathy [110]. This increase in COX-2 also reduces the amount of antiviral arachidonic acid, ultimately making patients more susceptible to COVID-19 and increasing their inflammation [111].

SARS-CoV-2 induces an increase in COX-2, HMGB1, RAGE, and GFAP levels in severe COVID-19 cases. Together, these biomolecules are correlated with neural dysfunction associated with hyperinflammation and high levels of ROS, and they could be used as possible indicators of severe cases of COVID-19 [ 109, 112] . The imbalanced ROS production seen in COVID-19 by the mononuclear phagocyte system (MPS) and neutrophils results in extensive neutrophil extracellular trap (NET) formation, inflammation, and tissue destruction. SARS-CoV-2 induces NET formation in otherwise healthy neutrophils. MPO-DNA and Cit-H3 are specific NET markers, which suggests that NET formation drives cytokine storms in COVID-19. ROS stimulates the NF-κB pathway to create TNF-α, IL-1, IL-8 and induce NET formation. Thus, the dysfunction of ROS homeostasis in the body seen in COVID-19 induces a cytokine storm [ 113, 114] .

## Various Antioxidants Used Prophylactically or Therapeutically in COVID-19

Antioxidants have been shown to be valuable as a complementary therapy for COVID-19 [115]. Quercetin, apigenin, baicalin, luteolin, hesperidin, genistein, proanthocyanidin, and eriodictyol have been reported to bind to SARS-CoV-2 protein components or the angiotensin-converting enzyme two receptors [ 116– 124] . For instance, quercetin, a flavonoid used predominantly as an antioxidant, is reportedly used as a prophylactic treatment for COVID-19 [ 55, 56] . Molecular docking studies indicate that quercetin has high binding affinities to various targets in SARS-CoV-2 [125]. A randomized controlled trial examined the impact of quercetin (1 g) in COVID-19 patients and found decreased severity of COVID-19 symptoms, duration of hospitalization, artificial ventilation, and fewer deaths in comparison with patients with standard care (without quercetin supplementation) [86]. Another pilot RCT found that 600 mg of quercetin improved COVID-19-related clinical symptoms and relevant plasma parameters [126]. Additionally, Chinese herbal remedies and polyphenolic compounds comprising antioxidants are used as an adjunct to lower the severity and mortality of COVID-19 patients [ 127, 128] . The various antioxidants with the potential to be used therapeutically or as a prophylactic measure in COVID-19 patients are summarized in Table 2.

**Table:** **Table 2** Antioxidants and their mechanisms against COVID-19

Antioxidant	Mechanism against COVID-19
Apigenin	*In-silico* studies indicate that apigenin could bind to S2 unit of spike protein (S) of SARS-CoV-2 [129].
Catechin	*In-silico* studies show binding to S protein of SARS-CoV-2 and hACE2, thus inhibiting viral entry [130].
Hesperidin	*In-silico* studies indicate that hesperidin may bind to multiple components of SARS-CoV-2 (like Mpro, PLpro, Spike protein) and its human receptor ACE2 [131].
Kaempferol	*In-silico* studies show that kaempferol can inhibit Spike glycoprotein of SARS-CoV-2 [132].
Luteolin	*In-silico* studies show luteolin binds strongly to Mpro, PLpro, and ACE-2. It can also bind to S2 unit of spike protein (S) of SARS-CoV-2 [ 120, 129] .
Myricetin	Inhibition of SARS-CoV-2 replication by targeting Mpro (in-silico) and ameliorating pulmonary inflammation [133].
Naringenin	*In-silico* evidence of Mpro inhibition and reduction of ACE-2 activity [134].
Quercetin	*In-silico* and *in vitro* studies demonstrates that quercetin can interfere with various stages of the coronavirus entry and replication cycle such as PLpro, 3CLpro, and NTPase/helicase [ 135, 136] .

## Effect of Currently Used COVID-19 Drugs on Oxidative Stress

Current guidelines for COVID-19 critical care include common supportive measures, such as hemodynamic support with a vasopressor (usually norepinephrine), corticosteroids to treat refractory shock, and mechanical ventilation to treat severe ards. furthermore, antivirals (remdesivir), antiparasitic agent (ivermectin), antibiotic (azithromycin), and anti-infective (hydroxychloroquine) have been utilized to decrease the viral replication and viral load. Monoclonal antibodies and anti-cytokine biologics to decrease the severity of the disease and vaccines to prevent the spread have been the mainstay of therapy against COVID-19. All these agents are known to modulate oxidative stress and thereby alter the natural course of the illness. The various agents that are implicated in the management of COVID-19 and how they modulate oxidative stress are discussed in Tables 3– 10, respectively.

**Table:** **Table 3** Effect of Corticosteroids ( *e*. *g*., dexamethasone) on oxidative stress

Effect of Corticosteroids on oxidative stress	Ref.
Increase markers of oxidative stress
Increase oxidative stress pathways at a dose of 0.2 mg/kg/7 days by increases of protein carbonyl, lipid peroxide, mitochondrial superoxide anions, and a decrease in sulfhydryl content in the cortex and hippocampus of pneumococcal meningitis infected rats.	[137]
Increase various markers of oxidative stress, such as the expression of heat shock protein 70 (Hsp70) and the content of 4-hydroxynonenal and nitro tyrosine in the cortex.	[138]
Antenatal betamethasone enhances the detrimental effects of postnatal dexamethasone on hyperoxic lung and brain injuries in newborn rats.	[139]
Increase generation of reactive oxygen species (ROS) and the activities of protease (calcium-dependent cysteine), calpain, and caspase-3. Also induce oxidative stress in osteoblast and hippocampal HT22 neurons, similar to effects in the human SH-SY5Y neuroblastoma cells.	[140]
Adrenalectomy increases the oxidative stress induced by 3-nitropropionic acid in the synaptosomes and exhibits neuroprotective effects.	[141]
Cause significant amyloid-β (Aβ) deposition, tau protein hyperphosphorylation, increased 4-hydroxynonenal content, and effects on cholinergic neurotransmission, similar to Alzheimer’s disease.	[142]
Decrease markers of oxidative stress
Act upon glucocorticoid receptors affecting heat shock protein 70 and induce downregulation of matrix metallopeptidase-9 (MMP-9) in eosinophilic meningitis-infected mice caused by *Angiostrongylus cantonensis*. Reduce expression of nuclear factor kappa B (NF-κB), c-Jun N-terminal kinase (JNK), and extracellular signal-regulated kinase (ERK) in the CSF and brain parenchyma. Decrease 8-hydroxy-2′-deoxyguanosine oxidized derivative (8-OHdG) content in the CSF.	[143]
Inducible NO synthase inhibition enhances the survival rate of *P*. *berghei*-infected mice.	[ 9, 117]
Melatonin exhibits antioxidant activity against dexamethasone-induced neurotoxicity in the human SH-SY5Y neuroblastoma cells.	[144]
Melatonin+dexamethasone combination shows synergistic neuroprotective effects in rodent models containing traumatic brain injury and intracerebral hemorrhage.	[145]
Radiation therapy in cancer causes cognitive impairment by inducing oxidative stress, mitochondrial dysfunction, neuroinflammation, and apoptosis. After radiation injury, a combination of vinpocetine+dexamethasone relieves cognitive impairment in nasopharyngeal carcinoma. This combination decreases TLR2, TLR4, IL-20, IL-8, TNF-α, IFN-γ, monocyte chemoattractant protein 2, and interferon expression. The combination also increases the antioxidant enzyme activities of superoxide dismutase, glutathione peroxidase, and glutathione reductase. Also, increase glutathione content and decreases prooxidant lipid peroxide.	[51]
Block organotin trimethyltin (TMT)-induced oxidative stress and apoptosis in adrenalectomized mice.	[146]
Increase the malonaldehyde and nitric oxide (NO) content and significantly decrease the antioxidant enzymes activities causing neurotoxicity in female rats.	[89, 147]
Inhibit brain superoxide dismutase activity and increase lactate dehydrogenase levels in male Wistar rats.	[148]
Exhibit neuroprotective effects against hepatic encephalopathy in rats.	[149]
Decrease oxidative stress by up-regulation of Nrf2, increasing antioxidant enzymes activities, and lowering lipid peroxidation in autoimmune encephalomyelitis (EAE) induced C57BL/6 mice treated with myelin oligodendrocyte glycoprotein 35-55 (MOG35-55).	[150]
Combination therapy with ciprofloxacin can produce antioxidant effects and protect against *S*. *aureus*-induced neurotoxicity caused by the induction of oxidative stress and brain abscesses leading to neurotoxicity.	[151]

**Table:** **Table 4** Effect of Antivirals ( *e.g*., remdesivir, lopinavir, ritonavir, saquinavir, darunavir, cobicistat, camostat mesylate) on oxidative stress

Effect of Antivirals on oxidative stress	Ref.
Increase markers of oxidative stress
Protease inhibitors (PIs), ritonavir, and saquinavir, alone or in combinations with the nucleoside reverse transcriptase inhibitor (NRTI), zidovudine (AZT), induce oxidative stress and neuronal damage/death in primary cultures at clinically relevant doses. Additionally, PI-induced oxidative stress and neuronal death in primary neurons can be inhibited by the activation of endogenous antioxidant response.	[152]
Antiretroviral-induced neurotoxicity has been shown in primary rat neurons.	[153]
Decrease markers of oxidative stress	
Remdesivir crosses the blood-brain barrier (BBB) to exhibit its pharmacodynamic effects in the brain.	[154]
Remdesivir inhibits SARS-CoV-2 infection of hiPSC neurons and astrocytes, suggesting ApoE4 may play a causal role in COVID-19 severity.	[155]
Remdesivir treatment exhibited an upregulation of tissue repair factors BDNF, PDGF-BB, PIGF-1, and an increased ratio of Th2-associated cytokine IL-4 to Th1-associated cytokine IFN-γ. There was a significantly higher magnitude of increase in Th2-associated IgG2 and IgG4 responses.	[156]

**Table:** **Table 5** Effect of Antiparasitic ( *e*. *g*., ivermectin) on oxidative stress

Effect of Antiparasitic on oxidative stress	Ref.
Increase markers of oxidative stress
Ivermectin-induced oxidative stress was identified as signs of significantly increased total erythrocyte count, packed cell volume, total leukocyte counts, and lymphocytes, as well as a large decrease in glutathione peroxidase and reduced glutathione.	[157]
Decrease markers of oxidative stress	
Ivermectin and ivermectin combined with a multi-walled carbon nanotube significantly decrease oxidative stress induced by spinal cord injury in Wistar rat subjects. Both treatments decrease pro-inflammatory cytokines (TNF-alpha, IL-1β, and IL-1) in the spinal cord and dorsal root ganglion tissues.	[158]
Subcutaneous administration of ivermectin with topical amitraz and antioxidants (Vit C, Vit E, and selenium) resulted in significantly decreased levels of lipid peroxidase that were originally elevated due to skin lesions in *Psoroptes natalensis* infected Indian water buffaloes. The activities of body antioxidants (GSH and CAT) were significantly higher after the treatment.	[159]
Suppressed cell proliferation by promoting ROS-mediated mitochondrial apoptosis pathway and inducing S phase arrest in colorectal cancer cells, suggesting the usage of potential anticancer drug therapy.	[160]

**Table:** **Table 6** Effect of Anti-infectives ( *e.g*., hydroxychloroquine, chloroquine) on oxidative stress

Effect of Anti-infectives on oxidative stress	Ref.
Increase markers of oxidative stress
Chloroquine treatment has been associated with significantly lower red blood cell levels of catalase (CAT) and GPX activities and increases of SOD1, which is an adaptive response to oxidative stress. Additionally, plasma levels of chemical antioxidants (Vit A, Vit C, GSH, beta-carotene) were significantly decreased, and malondialdehyde levels were increased, which is a measure of lipid peroxidation.	[89, 161, 162]
Chloroquine has also been associated with decreased serum GSH levels and increased MDA levels, indicating chloroquine-induced oxidative stress in animal models. Additionally, red blood cell levels of SOD1 were significantly increased, while red blood cell levels of CAT were significantly decreased.	[162, 163]
Acute chloroquine intraperitoneal injection increased NADPH-induced lipid peroxidation and decrease retinal tissue GSH content. Chronic chloroquine use did not induce NADPH-induced lipid peroxidation and increased retinal GSH content.	[162, 164]
Decrease markers of oxidative stress	
Hydroxychloroquine dosed 40mg/kg/day for 21 days reduced phorbol 12-myristate 13-acetate (PMA)-stimulated oxidant formation in the blood of rats with adjuvant arthritis. Hydroxychloroquine also decreases PMA-stimulated extracellular neutrophil oxidants but increases intracellular oxidant formation.	[162, 165]
Hydroxychloroquine significantly decreases the total levels of nitric oxide (NO), malondialdehyde, and reactive oxygen species (ROS) in human umbilical vein endothelial cells.	[89, 166]

**Table:** **Table 7** Effect of Macrolide antibiotic ( *e*. *g*., Azithromycin) on oxidative stress

Effect of Macrolide antibiotic on oxidative stress	Ref.
Increase markers of oxidative stress
Azithromycin blocked photosynthetic electron transfer causing electron accumulation and resulting in the formation of reactive oxygen species (ROS) and producing oxidative stress.	[167]
Decrease markers of oxidative stress	
Azithromycin has been associated with reducing the production of pro-inflammatory cytokines (IL-8, IL-6, TNF-alpha), reducing oxidative stress, and modulating T-helper functions.	[168]
Azithromycin decreases the secretion of IL-4, IL-5, IL-13, and IL-17A from peripheral blood mononuclear cells in patients with chronic obstructive pulmonary disease (COPD). It also decreases the production of IL-4 and IL-8 by CD4 ^+^ and CD8 ^+^ T cells. Additionally, azithromycin in combination with budesonide suppressed the inflammatory response by inhibiting IL-4, IL-5, IL-8, IL-13, IL-17A, IL-33, thymic stromal lymphopoietin (TSLP), macrophage migration inhibitory factor (MIF) release from peripheral blood mononuclear cells, and by reduction of the percentage of IL-4, IL-8, IFN-gamma, and TNF-alpha expressing CD4 ^+^ and CD8 ^+^ T cells.	[169]

**Table:** **Table 8** Effect of Anti-cytokine biologics ( *e*. *g*., tocilizumab, anakinra, baricitinib) on oxidative stress

Effect of anti-cytokine biologics on oxidative stress	Ref.
Increase markers of oxidative stress
No available evidence of increased oxidative stress.	–
Decrease markers of oxidative stress
Tocilizumab improved endothelial function leading to a greater increase of myocardial work than conventional synthetic disease-modifying antirheumatic drugs and glucocorticoids through a reduction of inflammatory burden and oxidative stress.	[170]
Tocilizumab showed evidence of altering genes that regulate mitochondrial dysfunction and oxidative stress through gene ontology analysis in neutrophils. In peripheral blood mononuclear cells, treatment of sJIA with *Tocilizumab* affected genes in oncostatin M signaling and B pathways.	[171]
The serum level of oxidative stress was lower in rheumatoid arthritis patients treated with Tocilizumab, suggesting IL-6 inhibition therapy reduces joint damage and vascular degeneration.	[172]
Anakinra decreased oxidative stress and endoplasmic reticulum stress when given as a single-dose morphine and tolerance induction in rats. It also decreased apoptosis proteins after tolerance development.	[173]
Anakinra improved vascular and left ventricular function through IL-1 inhibition, which was associated with reducing nitrooxidative stress and endothelin.	[174]

**Table:** **Table 9** Effect of Monoclonal antibodies ( *e.g*., bamlanivimab, etesevimab, sotrovimab, casirivimab, imdevimab) on oxidative stress

Effect of Monoclonal antibodies on oxidative stress	Ref.
Increase markers of oxidative stress
In monoclonal-treated mice with lung cancer, the tumor area and weight were significantly reduced, while T-cell counts, oxidative stress, apoptosis, autophagy, activated p65, and sirtuin-1 markers were increased.	[175]
Decreased markers of oxidative stress
Anti-Tn monoclonal antibody treatment improved hyperoxia-induced kidney injury in neonatal mice by decreasing kidney injury scores and cytokine levels. The advantage of anti-Tn monoclonal antibodies on hyperoxia-induced kidney injury is mediated by a decrease in oxidative stress, NF-kB expression, and increased IkB-alpha expression.	[176]
Many monoclonal antibodies used to treat inflammatory bowel disease (IBD) modify enzymatic activity, reduce oxidative stress, and downregulate pro-inflammatory transcriptional factors and cytokine secretion.	[177]

**Table:** **Table 10** Effect of Vaccines ( *e*. *g*., covishield, Pfizer-BioNTech, Moderna) on oxidative stress

Effect of Vaccines on oxidative stress	Ref.
Increase markers of oxidative stress
The relationship between oxidative stress and COVID-19 vaccine-induced heart involvement (acute pericarditis-myopericarditis is unknown. Low nitric oxide (NO) levels in myopericarditis indicate the inflammatory and procoagulant state in mRNA vaccine-induced myopericarditis. So, oxidative stress could be the role of mRNA vaccine-induced myopericarditis.	[178]
Decrease markers of oxidative stress
No evidence of a reduction of oxidative stress for COVID-19 vaccinations.	–

## Conclusions

Oxidative stress plays a crucial role in the pathogenesis of COVID-19 and perpetuates inflammation, cytokine storms, thrombosis, and neuronal dysfunction. An increase in prooxidants, namely, reactive oxygen and nitrogen species, along with decreased antioxidant defense systems are noted in SARS-CoV-2 infections. Various treatments, including nutraceuticals and conventional drugs, have been shown to decrease oxidative stress and offer substantial benefits in COVID-19 patients. However, the effect of various drugs, monoclonal antibodies, and vaccines on modulating oxidative stress in COVID-19 has been discussed. Further studies are required to investigate this issue to firmly use antioxidants as potential therapeutic agents in COVID-19 patients presenting with neurological manifestations.
